# Local public health projectification in practice: a qualitative study of facilitators and barriers to a public health plan implementation

**DOI:** 10.1177/14034948221080402

**Published:** 2022-03-18

**Authors:** Heidi M. Nilsen, Eli Feiring

**Affiliations:** Department of Health Management and Health Economics, University of Oslo, Norway

**Keywords:** Public health, public sector, projectification, trans-sectoral collaboration, implementation, public planning, organisations, Public Health Act, Norway

## Abstract

**Aims:**

To identify factors perceived by local government employees to affect the implementation of a municipal public health plan.

**Methods:**

Qualitative individual interviews (*n*=13) were carried out. Participants were sampled from three districts in Oslo municipality, Norway, and asked about their experiences with an ongoing implementation of the Oslo Public Health Plan (2017–2020). The conceptual framework of public sector projectification – a growing reliance on project organisation of policies – informed the study. The consolidated framework for implementation research was used to aid data coding and subsequent thematic analysis.

**Results:**

Implementation facilitators included factors perceived to support flexibility, including plan adaptability to the local setting, and factors perceived to enable structure and control during the implementation process, such as the articulation of specific goals and a shared understanding of public health work. Barriers were mainly related to complex aspects of the plan, such as the need to involve multiple stakeholders and levels of governance, and to tensions between the time-limited implementation process and the permanent organisational structures.

**Conclusions:**

**This study has demonstrated how research-based methods can be used for the evaluation of a local community implementation process. It has identified implementation determinants using a predetermined taxonomy of operationally defined factors that are likely to influence implementation. However, while implementing a time-limited public health plan can be seen as ‘taking action’ in relation to multidimensional and complex problems, further research is needed to investigate whether plan implementation has a long-term impact on the surrounding organisation and, eventually, on public health outcomes.**

## Background

In May 2017, the municipality of Oslo launched the Oslo Public Health Plan 2017–2020 [1]. The realisation of the plan’s intended effects on improving citizen’s health and wellbeing, irrespective of who they are and where in Oslo they live, is closely linked to plan implementation within the 15 districts of the municipality.

However, public health interventions designed to improve overall health in society and reduce health inequities are not easy to implement [2–6]. Such interventions are characterised by a population focus, and take place in the community, drawing on an expansive interdisciplinary evidence base encompassing multiple and interrelated factors and long and complex causal chains [7]. Furthermore, many public health interventions require trans-sector governance anchored in a ‘health in all policies’ (HiAP) approach [8–10]. These unique features give rise to specific challenges to successful implementation of public health interventions. Barriers include parallel and conflicting understandings of public health, disagreements in areas of priority and ways to execute the work, and difficulties in establishing and sustaining cooperation between organisations that perhaps do not find the goal of the HiAP relevant to them [11].

It is strongly recommended that public health governance is committed to a whole-of-government and whole-of-society approach [12]. This relates both to the vertical, multilevel relationships between governance at national and local levels, as well as the horizontal cross-sectional aspects of governance, involving multiple actors. To manage risk better and create more enduring policies, decentralising decision making to the local level is suggested. At the same time, public organisations may find it tempting to address some of the complexities of public health governance by the use of short-term projects. Developing and implementing a public health plan can be understood as a means used by decision makers to combat some of the implementation challenges described above. A plan formulates an appealing vision of public health, specifies aims and tools, and can direct the organisation’s attention to public health work, thereby aiding policy making. Moreover, the implementation process itself, organised through the means of goal setting, deadlines, management, teams and designated resources, represents a project logic.

However, the short-term nature of projects may seem to run counter to the long-term policy objectives. Critics of public policy projectification – that is, the growing reliance on project organisation of policies, have pointed out that project organisation offers fragmented and unsustainable short-term solutions [13]. Moreover, responsibility is delegated from ordinary administration structures and roles to time-limited project management and teams, which can be seen as a depoliticisation of policies. How these processes actually unfold in practice is not well known.

In this study, we used the theoretical perspective of public health projectification to gain better knowledge of a public health plan implementation. By focusing on characteristics of the plan, the process, the local organisation and the wider context, the study sought the perspectives of employees working with local public health initiatives on factors likely to be critically important to plan implementation.

## Aims

This study aimed to investigate factors perceived by local government employees to affect the implementation of the Oslo Public Health Plan through the lens of public sector projectification (Table I) [14, 15].

**Table I.** Study setting, adapted from Baldersheim et al. [14] and Folkehelseloven (Public Health Act) [15].

Administrative levels	Central state Counties Municipalities
Norwegian Public Health Act	Based on the principle of health equity and the HiAP approach
Municipal autonomy	Considerable financial and organisational autonomy
Municipal responsibility according to the Norwegian Public Health Act	Extensive range of responsibilities, including: promotion of the population’s health and wellbeing; protection against factors that may impact the population’s health negatively, and contribution to reduced social inequalities in health. The municipality should do this by trans-sector cooperation, developing overviews of citizen’s health and health determinants in order to prioritise which public health concerns should be incorporated in the local planning systems, so that appropriate measures can be executed.

HiAP: health in all policies.

## Methods

### Conceptual frameworks

Theoretical perspectives can be used to guide data collection, analysis and interpretation, and make us able to build on findings across studies and contexts [16]. Public sector projectification was used as a theoretical perspective to guide the present study.

Public sector projectification denotes an approach to public administration and management in which work is organised in a series of projects alongside routine public services activities [13].

Two seemingly contradictory features of the project logic are observed [17]. On the one hand, the project logic is used to support common values, cooperation and organisational change. On the other, the project logic strengthens predictability and control using project management hierarchies, procedures, funding and evaluation. Thus, organising work in projects is perceived simultaneously to enable flexibility and control, a duality perceived as attractive by decision makers. However, critics have pointed out how greater fragmentation of political decision making can result in depoliticisation when too much responsibility for policy outcomes is delegated to project management, and worry that functionalist concerns of projects’ effectiveness and efficiency gain importance at the expense of other relevant concerns [13].

In this study, we used projectification to help us choose which implementation determinants to look for and specify the categories in which factors were to be grouped. In understanding the implementation of the public health plan, we focused on the intervention characteristics and implementation process of the given plan (the ‘project’), and we furthermore assumed that the organisation’s inner and outer settings were likely to influence implementation.

To provide a more granular understanding of the factors and conceptualise subfactors that can potentially affect implementation outcomes, we supplied the projectification perspective with the consolidated framework for implementation research (CFIR) [8] (Table II). CFIR is a meta-theoretical determinant framework that describes 39 theoretical constructs across five contextual domains. The framework enables systematic and comprehensive identification of potential explanatory themes that are likely to influence implementation. By linking the theoretical perspective of projectification to CFIR, we sought to identify factors most salient to implementation of the public health plan.

**Table II.** CFIR domains and constructs: overview adapted by Damschroder et al. [18].

Domain	Constructs
Intervention characteristics	Intervention source, evidence strength and quality, relative advantage, adaptability, trialability, complexity, design quality and packaging, cost
Outer setting	Needs and resources of those served by the organisation, cosmopolitanism, peer pressure, external policy and incentives
Inner setting	Structural characteristics, networks and communications, culture, implementation climate: tension for change, compatibility, relative priority, organisational incentives and rewards, goals and feedback, learning climate, readiness for implementation, leadership engagement, available resources, access to knowledge and information
Characteristics of individuals	Knowledge and beliefs about the intervention, self-efficacy, individual stage of change, individual identification with organisation, other personal attributes
Process	Planning, engaging: opinion leaders, formally appointed internal implementation leaders, champions, external change agents, executing, reflecting and evaluating

CFIR: consolidated framework for implementation research.

### Study participants

We used a snowball sampling technique by asking the Oslo Agency for Health to inform public health contacts of the study in all 15 districts of Oslo, and to distribute invitation letters. Three districts were willing to participate and identified relevant employees. We wanted participants that worked for the organisation with the development and implementation of public health initiatives, including the public health plan, such as coordinators, project managers and team leaders. Thus, we sought to recruit participants purposively to gain insight into the implementation process [19].

### Data collection procedures

In-depth individual interviews were carried out by the first author at the participants’ workplace during spring 2018. The average length of the interviews was 40 minutes. The interviews were semi-structured following a topic guide with open-ended questions that covered themes such as conceptualisation of public health, organisation of the public health work in the district, facilitators and barriers to local imple-mentation of the Oslo Public Health Plan, and reflections around the implementation process. The interviews were recorded on a password protected smart phone and uploaded to a university server-connected computer, and subsequently transcribed by the interviewer.

### Data analysis

A step-by-step guide [20] was used to conduct a thematic analysis. First, the data were read and re-read. Then, data were coded thematically according to four of the five CFIR domains (themes) and constructs (subthemes) [18]: intervention characteristics; implementation process; inner setting; and outer setting. The fifth domain, individual characteristics, was not applied because we did not aim to investigate individual-level change.

Frequency tables were kept while coding to assess salience. This is aligned with the recommendations by Damschroder et al. [18], because it includes a quantitative aspect to the analysis that help in making the most out of the collected data [21, 22]. The determinants that were less frequent were excluded or merged with other codes into broader themes during refinement of the analysis. The themes and subthemes were then revised in relation to the original data to confirm that they captured the essential meanings. Both authors participated in data coding and discrepancies were discussed until agreement was reached. Citations to exemplify arguments were chosen and translated from Norwegian to English by the authors.

### Ethics

The study was notified to the Norwegian social science data services (project no. 57949) and deemed to be in accordance with relevant guidelines. The participants were informed of the voluntary nature of their participation and signed an informed consent before the interviews began. All quotes used in the article were read through and approved by the participants.

## Results

Three large-population districts accepted the invitation to participate. We did not receive information about why the other districts did not respond to the invitation. The districts that participated are located in the eastern, central and western parts of Oslo. They have different socioeconomic profiles, where the population in the western districts on average has a longer educational background and fewer low-income households compared to other parts of the municipality [23]. As health status is related to levels of income and educational background, there are social inequalities in health not just across the districts but also within them [1].

All employees formally appointed within the organisation to work with the implementation of the public health plan in their district were invited to participate in the study; all accepted the invitation (*n*=13). The participants differed in their academic backgrounds and roles. Districts A and B each provided four participants, while district C provided five (Table III).

**Table III.** Interview participants.

Role	Number
Public health coordinator (folkehelsekoordinator)	3
Coordinator for the voluntary sector (frivillighetskoordinator)	1
Head of health-related department (avdelingsleder, helse)	2
Dietician (klinisk ernæringsfysiolog)	1
Chief district medical officer (bydelsoverlege)	1
Head architect (sjefsarkitekt)	1
Advisor, care services (rådgiver, omsorgstjenester)	2
Senior executive officer, planning and building (konsulent, plan og bygg)	1
District director (bydelsdirektør)	1
Total	13

Table IV exemplifies participants’ quotes and Table V summarises the results.

**Table IV.** Examples of participants’ quotes.

Domain	Constructs	Illustrative quotes
Intervention characteristics	Complexity and adaptability	I have to do a ‘translation’ in order to make it (the plan) useful for my employees. (District director)
	Relative advantage	So many plans overlap (. . .). Having that many municipal plans to incorporate makes the implementation all the way out to the actual services much harder, because eventually people don’t relate to it (. . .). If you consider public health as being overarching and pervasive in everything, then perhaps it would be sufficient with a public health plan. (Head of department 1)
Implementation process	Planning	We got really positive feedback, so it (seminar in the district about the implementation of public health plan) went really well. But it could’ve been a disaster if we wouldn’t have been able to convey the content effectively. (Public health coordinator 2)
	Opinion leaders and implementation leaders	You have to knock on closed doors many times over and fight pretty hard in order to gain legitimacy in the organisation and the authority you need in order to practice your discipline. (Public health coordinator 2)
	Engaging the public	Public participation is important, not just to get suggestions, but because if you manage to do it right, it can provide as sense of empowerment to the ones that participate. So that is also a way of working with public health: activating our own community. (Public health coordinator 1)
Inner setting	Structural characteristics, networks and communications	We’ve been very concerned with the fact that we’ve been working too much in ‘silos’ in this district, which is perhaps also true for Oslo municipality in general; all of these agencies – that are important for public health – that don’t cooperate. (Advisor, care services 1)
	Compatibility and relative priority	That’s what’s so challenging about public health; when it ‘burns’ elsewhere, public health is hard to prioritise. (Public health coordinator 1)
	Readiness for implementation	If it is assumed that public health is all about diet and exercise, it will be hard to implement traffic safety measures, for instance. Not establishing a mutual understanding of the public health plan (. . .) could make the implementation of it more difficult. (Public health coordinator 1)
	Leadership engagement	I have to create legitimacy for the (public health) work. The public health coordinator ‘acts’ on my behalf. (District director)
	Available resources	If there is too big a gap between action plans and economy, then I believe that ‘public health’ won’t be anything but a piece of paper. (Chief district medical officer)
Outer setting	Needs and resources of those served by the organisation	We’ve made a local health overview (. . .), which puts focus on the health state in district’s population (. . .). We’re going to work with those areas that the statistics indicate are our biggest challenges. (Coordinator for the voluntary sector)
	External policy and incentives	It (public health) has become ‘trendy’. Public health is, in general, a field that has benefited a lot by introducing national guidelines through the Public Health Act. (Public health coordinator 1) The Public Health Act mentions the responsibility of county authorities and municipalities. But what’s the responsibility of the districts? Okay, so the Public Health Act hasn’t covered this, but then the municipality of Oslo should clarify what is expected as a minimum of the districts. (. . .). If I fail to ‘promote’ public health as an area of priority to my leader team, then somebody can just ‘cut through’ and say: ‘We’re not going to prioritise this’ because nothing requires us to. (Public health coordinator 2)

**Table V.** Summary of findings: facilitators and barriers.

Domain	Constructs	Facilitators	Barrier
Intervention characteristics	Complexity		x
	Adaptability	x	
	Relative advantage		x
Implementation process	Planning	x	
	Opinion leaders	x	
	Implementation leaders	x	
	Engaging the public	x	
Inner setting	Structural characteristics		x
	Networks and communications		x
	Compatibility with existing processes		x
	Relative priority		x
	Readiness for implementation	x	
	Leadership engagement	x	
	Available resources		x
Outer setting	Needs and resources of those served	x	
	External policy and incentives	x	x

### Intervention characteristics domain

#### Intervention complexity, adaptability and relative advantage

The plan was perceived as extensive, with a high number of planned actions that varied greatly in terms of detail level. Every participant viewed the implementation of the plan as a complex process and reported that the plan needed some adaptation to the local setting. The participants proposed alternative ways to organise the municipal planning for public health in Oslo, such as integrating plans relevant for public health into one plan.

### Implementation process domain

#### Planning, leaders and public engagement

Participants recognised the importance of careful planning of the implementation process. Having designated public health coordinators and other public health-related positions that were responsible for putting pressure on the implementation process and anchoring public health across sectors was described as a valuable resource. It was described as important to engage the public in the process to meet the needs of the population in the district.

### Inner setting domain

#### Structural characteristics, networks and communications and compatibility with existing processes and relative priority

The structural characteristics of the municipality of Oslo were among the most frequently mentioned themes when discussing barriers to implementation of the public health plan. The organisational structure was described as ‘complex’ and ‘fragmented’. This was perceived as a challenge to trans-sectoral cooperation, which was perceived as essential for public health work by the participants. Several of the participants thought that other initiatives in the municipality were given higher priority than public health.

#### Readiness for implementation and leadership engagement

The participants perceived developing knowledge about public health and a common understanding of public health work in the organisation to be essential in implementing the plan. A continuous focus and pressure from the top management was pointed out as important for successful implementation.

#### Available resources

The participants explained how their respective districts had increased the resources spent on public health by creating positions and start-up projects. Lack of resources was, however, still reported in most interviews.

### Outer setting domain

#### Needs and resources of those served by the organisation, external policy and incentives

Participants described addressing the needs of those living in the district as essential and discussed how to gain knowledge about their health status. Putting together health overviews was mentioned as an important tool to achieve this. The public health field was described as ‘up and coming’, and that there was an overall willingness and awareness around public health that had not previously been present. National policies were perceived to aid local engagement, although some of the participants wished that the Public Health Act specified the role of the districts.

## Discussion

Public health work in Norway has become increasingly anchored across sectors [24, 25]. The results of the present study illustrate this, as participants emphasised a focus on trans-sectoral collaboration as essential for successful implementation of the public health plan.

However, trans-sectoral work may pose specific challenges. Previous research shows that a major barrier to public health work is the existence of parallel and conflicting understandings of what public health entails [2–6]. A project logic can offer a common language and a shared vision of goals. The participants in our study perceived a shared understanding of public health in the district administration as a prerequisite for implementing the public health plan. This finding aligns with previous studies on enabling public health work [9, 10].

Two seemingly contradictory factors were both seen as facilitators. On the one hand, local adaptation of the plan was perceived as a factor enabling implementation. On the other, strong leadership engagement from the top management in the municipality was viewed as a facilitator, including the opportunity to mobilise upper agencies for trans-sectoral cooperation with the districts, and to clarify the districts’ responsibilities according to the Public Health Act. These findings mirror research showing that local implementation of public health policy needs to be anchored at the executive and administrative level in the municipalities to be properly implemented [24]. An earlier study found that having public health coordinators can have positive effects on implementing HiAP [26], which was also identified as an important facilitator to implementing the public health plan in this study. Taken together, these findings illustrate how the duality of projectification was found to be attractive to the participants. The implementation of the plan offered flexibility through local adaptability, yet at the same time strengthened chains of command through project development and steering.

The participants talked about how the public health field is overarching and extensive, and therefore demands collaboration across sectors in a way that is hard to incorporate into existing bureaucratic structures. The structural characteristics of Oslo municipality were perceived to hinder plan implementation, and some of the participants expressed the idea that the plan itself was a manifestation of the organisational distance between the central and local levels of government. The plan was described as overlapping and sometimes incompatible with other municipal and local plans in the districts. The result was a plan that was perceived as complex. Such factors have been identified by others as barriers to policy implementation [9, 27].

Establishing networks, communication and an organisational culture related to public health work were salient examples of how the participants perceived the most important facilitators to the local implementation of the public health plan as also being the biggest barriers. This finding is not surprising, given the literature on projectification: while project organising is seen as a response to a need to solve tasks that have a high degree of complexity and interdependence between actors, and offer a chance to learn from a smaller setting before broadening the action, there is a risk that the project will not succeed in achieving change after it is terminated [28]. Managing trans-sectoral collaboration is a known challenge in public health work because it is difficult to establish and sustain [9–11]. The purpose of the Norwegian Public Health Act [15] is to contribute to societal development that promotes public health and reduces social inequalities in health. This is dependent on long-term ‘whole-of-society’ efforts across sectors [12], which could be argued makes it a bad fit for a project organisation.

Others have studied how a project organisation, despite being embedded in the permanent organisation’s activities, cultures, structures and processes, may encounter challenges due to diverging interests and shifting responsibilities [29]. While the intention may be to involve actors from different professions and organisations that otherwise do not work together, thus contributing to change and renewal, the institutional logics may not be easy to integrate. These factors must be better understood to overcome barriers related to the relationships between the temporary project organisation and the permanent organisations and to enable long-term and lasting impacts of implementing a time-limited public health plan.

### Implications for practice and theory development

The present study shows how research-based methods can be used for local government implementation processes and may inform practitioners that carry out implementation of innovation in other contexts. Moreover, the study contributes to theory development by indicating how public sector projectification can be supplied by a determinant framework to refine the conceptualisation of projectification. The use of CFIR offers a common language by which determinants of implementation can be articulated and a standardised list of constructs that can be used to identify salient variables [30]. Further studies are needed to promote theoretical development.

### Limitations

The use of CFIR, a predefined framework, may have led us to overlook themes [18]. However, the interview guide consisted of broad open-ended questions that covered relevant themes for the study. Further, we did not systematically investigate the individual characteristics domain, because we chose an organisational and structural focus for analysis. Moreover, identifying single constructs as facilitators and barriers was difficult, as many of the constructs are interrelated and intertwined. This is, however, a limitation that follows from the choice of a menu-of-constructs approach [21].

As this was a during-implementation study, we were not able to assess associations between constructs and outcomes.

We acknowledge that the small number of participants is a limitation of the study. Data were elicited from participants recruited from three out of 15 districts in the municipality. We do not know why the other districts did not respond to the invitation to participate in the study. Moreover, as other stakeholders such as policy makers and the public were not included, the transferability of findings may be limited.

Data were collected early in the implementation process (2018). A similar study at a later stage may yield valuable information about whether the participants’ experience with implementation of the Oslo Public Health Plan 2017–2020 will be different.

## Conclusions

This study has identified factors perceived by local government employees to affect the local implementation of the Oslo municipality Public Health Plan 2017–2020, using public policy projectification as a theoretical lens. We supplied the analysis with the use of CFIR to get a more granular understanding of implementation determinants and demonstrated how research-based methods can be used for evaluation of local community implementation processes. By utilising a determinant framework, this study has suggested how researchers and practitioners can articulate factors that potentially affect implementation of public health initiatives. Factors both enabling flexibility and innovation in the organisation, and predictability and control, were simultaneously perceived to facilitate implementation. Barriers included a tension between a project logic, requiring networked organisation and extensive collaboration, and established organisational structures. Most importantly, the inherent complexity of trans-sectoral collaboration for public health was seen as a significant threat to successful public health plan implementation.
